# Impulse oscillometry in the diagnosis of cough variant asthma in children

**DOI:** 10.1186/s12887-024-04749-4

**Published:** 2024-05-03

**Authors:** Chunyu Tian, Shiqiu Xiong, Shuo Li, Xin Song, Yantao Zhang, Xinmei Jiang, Xinyue Hou, Yifan Zhang, Chuanhe Liu

**Affiliations:** Department of Allergy, Children’s Hospital Affiliated with the Capital Institute of Pediatrics, No.2 Yabao Road, Chaoyang District, Beijing, 100020 China

**Keywords:** Impulse oscillometry, Cough variant asthma, Diagnosis, Children

## Abstract

**Background:**

Cough variant asthma (CVA) is one of the most common causes of chronic cough in children worldwide. The diagnosis of CVA in children remains challenging. This study aimed to assess the diagnostic utility of impulse oscillometry (IOS) pulmonary function in children with CVA.

**Methods:**

This study included children aged 4 to 12 years diagnosed with CVA who underwent IOS pulmonary function and bronchodilation (BD) tests. A control group of healthy children was matched. Pre- and post-BD IOS parameters were recorded and presented as mean ± standard deviation or median. Receiver operating characteristic (ROC) curves were plotted, and the area under the curve (AUC) was calculated to evaluate the discriminatory potential of the IOS parameters for diagnosing CVA.

**Results:**

A total of 180 patients with CVA and 65 control subjects were included. The baseline IOS parameters in the CVA group, except X5%pred, were significantly greater compared to the control group. After inhalation of salbutamol sulfate, all IOS parameters improved significantly in the CVA group. However, Z5%pred, R5%pred, and R20%pred remained greater in the CVA group compared to the control group. The improvement rates of IOS parameters in the CVA group significantly surpassed those in the control group. The ROC curve results for pre-BD IOS parameters and the improvement rate during the BD test showed that the combinations of pre-Z5%pred+_△_Z5% and pre-R5%pred+_△_R5% achieved the highest AUC value of 0.920 and 0.898, respectively. The AUC values of these combined parameters surpassed those of individual ones.

**Conclusions:**

This study highlights that children with CVA exhibit greater IOS parameters compared to healthy children. The changes in IOS parameters during the BD test provided valuable diagnostic information for CVA, and the combination of various parameters can help pediatricians accurately identify CVA in children.

## Introduction

The prevalence of chronic cough in Europe and the USA ranges from 5 to 33% [1, 2], and in recent years, there has been an annual increase in children [3]. Cough variant asthma (CVA) is one of the most common causes of chronic cough worldwide [4, 5], affecting 41.95% of children with chronic cough in China [3]. According to the third nationwide survey of childhood asthma in urban areas of China, CVA accounted for 9.7% of asthma [6]. Considered a milder subtype of asthma, CVA shares many pathophysiological characteristics with classic asthma, including atopy, airway eosinophilic inflammation, responsiveness to anti-asthmatic drugs, and relatively milder bronchial hyperresponsiveness (BHR) [7, 8]. Additionally, approximately 30% of CVA patients progress to classic asthma without inhaled corticosteroids (ICS) [9, 10]. Therefore, early identification of CVA is crucial for making clinical decisions and improving prognosis.

Nonetheless, the diagnosis of CVA in children remains challenging [11]. 2023 GINA MAIN REPORT only mentions how to differentiate CVA from eosinophilic bronchitis [12]. The Chinese national guidelines for the diagnosis and management of cough (2015) do not provide specific diagnostic criteria for CVA but recommend diagnosing CVA based on BHR and responsiveness to treatment [13]. The bronchial provocation test (BPT), a primary method for assessing BHR, is complicated and time-consuming. Moreover, it requires children’s cooperation and increases the risk of severe bronchospasm [14], thus limiting its use in children. The diagnostic method based on therapeutic efficacy in children is still debated [12] and may result in over- or underdiagnosis [15, 16]. Spirometry and bronchodilation(BD) tests are convenient and safe for evaluating airflow limitation in children [15]. Additionally, they are essential for determining the cause of chronic cough in children [11]. However, the diagnostic criteria for asthma, such as an improvement rate of FEV_1_ ≥ 12%, are not suitable for identifying CVA, as children with CVA often exhibit normal or near-normal pulmonary function and mild improvement after inhalation of bronchodilators [5, 12, 17]. Consequently, identifying a new method for the early diagnosis of CVA in children and distinguishing it from other causes of chronic cough is imperative.

Recently, impulse oscillometry (IOS) has emerged as a popular method for assessing children’s lung function [18]. This test, conducted during tidal breathing, is particularly suitable for preschool children who are unable to complete a maximal expiratory flow-volume curve. More importantly, the IOS can discriminate between dysfunctions in the small and large airways and is more sensitive than spirometry in detecting peripheral airway obstruction [19]. A recent study has revealed that combining fractional exhaled nitric oxide (FeNO) with IOS could yield significant efficacy in distinguishing CVA from chronic cough among preschool children [20]. Nevertheless, standardized criteria for IOS pulmonary function and BD tests in CVA children across a broader age spectrum are currently lacking. Therefore, this study aimed to investigate the changes in IOS parameters and BD test results in children with CVA and explored the diagnostic value of IOS pulmonary function in this population.

## Methods

### Study design and population selection

This single-center cross-sectional study was conducted at the Department of Allergy, the Children’s Hospital Affiliated with the Capital Institute of Pediatrics from March 2019 to January 2020. All parents and children involved in this study signed a written informed consent form. This study protocol was approved by the Institutional Ethics Committee(Approval number: SHERLL2019015). The inclusion criteria comprised patients aged 4 to 12 years who were diagnosed with CVA based on the guidelines for the diagnosis and optimal management of asthma in children (2016) [17]. The exclusion criteria included children with respiratory infections in the past four weeks, other chronic respiratory diseases (e.g., pulmonary tuberculosis, cystic fibrosis, bronchopulmonary dysplasia, classical asthma), or other diseases (e.g., congenital heart disease and chest malformations). Additionally, children receiving any anti-asthmatic drugs (e.g., inhaled or systemic corticosteroids and bronchodilators) were excluded. Healthy children aged 4 to 12 years were included as the control group.

### Data collection

Demographic characteristics such as sex, age, height, weight, and medical history of enrolled children were collected. The IOS test was performed, comprising baseline pulmonary function and BD tests. Main IOS parameters, including respiratory impedance at 5 Hz (Z5, kPa/L/s), resistance at 5 Hz (R5, kPa/L/s), resistance at 20 Hz (R20, kPa/L/s), reactance at 5 Hz (X5, kPa/L/s), integrated area of low-frequency X (AX, kPa/L), and resonant frequency (Fres, 1/s), were extracted. The value of R5-R20(kPa/L/s) was derived by subtracting R20 (kPa/L/s) from R5(kPa/L/s). Additionally, Z5, R5, R20, and X5 were analysed as a percentage of their actual values to their predicted values. R5-R20, AX, and Fres were analysed using their raw values. The bronchodilator response (BDR) of Z5 was calculated using the formula: BDR=(Z5_post_ − Z5_pre_)/Z5_pre_. The BDR for the other parameters was calculated using the same formula.

### IOS pulmonary function and bronchodilation test

In this study, IOS tests were conducted using a Mastscreen pulmonary function instrument (Jaeger, Germany) according to the ERS Task Force recommendations [21]. The percent predicted values of IOS parameters were calculated based on the equations published by Nowowiejska et al. [22]. Briefly, the IOS pulmonary function instrument was calibrated daily. During the measurements, all subjects were seated and instructed to breathe normally through the mouthpiece while their nose was clamped with a nose clip and their cheek was supported by the operator’s hand. All subjects were instructed to provide at least three technically acceptable measurements, each lasting 30 s, and the average of three measurements was recorded for analysis. Following the baseline test, the subjects inhaled 0.2% salbutamol sulfate (GlaxoSmithKline, UK) via a PARI compressor nebulizer with a mask at a dose of 1.25 ml for children younger than six years of age or 2.5 ml for those older than six years. The IOS test was repeated at 15-minute intervals after nebulization.

### Statistical analysis

The data were analyzed using SPSS 22.0 software (SPSS Inc., Chicago, IL, USA). Categorical data were summarized as percentages(%), whereas continuous data were presented as mean ± standard deviation or median (quartile 1, quartile 3). For categorical variables, the chi-square (χ^2^) test was used to determine statistical significance. The continuous data between the control and case subjects were compared using the two independent t-test or the Mann-Whitney U test. The continuous data before and after the BD test were compared using the paired sample t-test or the Wilcoxon matched pairs test. The receiver operating characteristic (ROC) curve was generated for each parameter of IOS, as well as for the combination of each parameter and its corresponding improvement rate during the BD test. The maximum of the Youden index was used to determine the optimal cutoff value of the IOS pulmonary function parameters for diagnosing CVA in children. Moreover, The sensitivity, specificity, and area under the ROC curve (AUC) were calculated to assess the ability of the parameters of IOS pulmonary function in recognizing CVA. Statistically significant differences were considered when the two-tailed p-value was less than 0.05.

## Results

### Basic characteristics

A total of 180 patients with CVA and 65 control subjects were included in this study. The mean age of the 180 patients was 5.5 [5.1, 6.8] years, and 53.3% were female. The mean age of the control group was 5.5 [5.0, 7.0] years, and 40.6% were females. The body mass index (BMI) was 15.72[14.36, 17.31] kg/m^2^ in the CVA group and 15.45[14.42, 17.13] kg/m^2^ in the control group. There were no significant differences in age (*P* = 0.183), sex (*P* = 0.081), or BMI (*P* = 0.687) between the two groups.

### Pre- and post-BD IOS pulmonary function parameters in the CVA group

As presented in Table 1, significant improvements in all IOS parameters were observed in the CVA group after inhaling salbutamol sulfate. These improvements encompassed Z5%pred, R5%pred, R20%pred, R5-R20, X5%pred, AX, and Fres (*P* < 0.05).

**Table 1:** Pre- and post-BD IOS pulmonary function parameters in the CVA group

	CVA group			
	pre-BD	post-BD	T/Z	P
Z5(%pred)	111.36 ± 27.60	86.29 ± 21.49	21.532	< 0.001
R5(%pred)	110.64 ± 27.89	86.27 ± 22.16	19.987	< 0.001
R20(%pred)	89.26 ± 20.11	76.08 ± 17.50	10.876	< 0.001
R5-R20(kPa/L/s)	0.32 ± 0.18	0.20 ± 0.14	13.616	< 0.001
X5(%pred)	116.46 ± 29.96	85.97 ± 27.30	16.076	< 0.001
AX(kPa/L)	2.31[1.54,3.31]	1.24[0.74,1.81]	-11.244	< 0.001
Fres(1/s)	20.09[18.18,22.40]	17.36[15.12,19.09]	-10.100	< 0.001

Abbreviation: Z5: respiratory impedance at 5 Hz; R5: resistance at 5 Hz; R20: resistance at 20 Hz; X5: reactance at 5 Hz; AX: integrated area of low-frequency X; Fres: resonant frequency

### Pre and post-BD IOS pulmonary function parameters in the CVA and control group

As shown in Table 2, all baseline IOS parameters (Z5%pred, R5%pred, R20%pred, R5-R20, AX, and Fres), except X5%pred, were significantly higher in the CVA group compared to the control group (*P* < 0.05). After the inhalation of salbutamol sulfate, Z5%pred, R5%pred, and R20%pred exhibited significant improvements in the CVA group compared to the control group (*P* < 0.05) (Table 2). However, R5-R20, X5%pred, AX, and Fres showed no significant differences between the two groups during the BD test.

**Table 2:** Pre-BD IOS pulmonary function parameters in the CVA group and control group and post-BD IOS pulmonary function parameters in the CVA group and control group

	pre- BD				post- BD			
	CVA group	control group	T/Z	P	CVA group	control group	T/Z	P
Z5(%pred)	111.36 ± 27.60	86.44 ± 16.67	-8.514	< 0.001	86.29 ± 21.49	77.76 ± 15.78	-3.240	0.002
R5(%pred)	110.64 ± 27.89	84.94 ± 16.89	-8.632	< 0.001	86.27 ± 22.16	77.30 ± 15.96	-3.345	< 0.001
R20(%pred)	89.26 ± 20.11	72.20 ± 14.45	-6.763	< 0.001	76.08 ± 17.50	67.67 ± 13.12	-3.869	< 0.001
R5-R20(kPa/(L/s)	0.32 ± 0.18	0.21 ± 0.13	4.209	< 0.001	0.20 ± 0.14	0.18 ± 0.11	0.934	0.351
X5(%pred)	116.46 ± 29.96	94.06 ± 21.67	-5.527	< 0.001	85.97 ± 27.30	82.48 ± 20.26	-0.851	0.396
AX(kPa/L)	2.31[1.54,3.31]	1.61[0.83,2.14]	-4.998	< 0.001	1.24[0.74,1.81]	1.02[0.67,1.64]	-1.196	0.232
Fres(1/s)	20.09[18.18,22.40]	18.61[15.52,19.72]	-4.954	< 0.001	17.36[15.12,19.09]	17.09[14.66.18.69]	-0.667	0.505

### Comparison of the improvement rate of the main IOS pulmonary function parameters between the CVA group and control group

As illustrated in Table 3, the improvement rates of all the IOS parameters (_△_Z5%, _△_R5%, _△_R20%, _△_R5-R20%, _△_X5%, _△_AX%, and _△_Fres%) within the CVA group significantly surpassed those in the control group (*P* < 0.05).

**Table 3:** Comparison of the improvement rate of the main IOS pulmonary function parameters between the CVA group and control group

	CVA group	control group	T/Z	P
_△_Z5(%)	-21.90 ± 10.89	-5.99 ± 9.53	9.290	< 0.001
_△_R5(%)	-21.13 ± 12.12	-6.43 ± 8.96	7.981	< 0.001
_△_R20(%)	-16.07[-23.15, -4.98]	-2.56[-11.85, 4.32]	-4.642	< 0.001
_△_R5-R20(%)	-38.68[-57.02, -18.60]	-22.65[-51.05, 0.00]	-2.378	0.017
_△_X5(%)	-24.31 ± 17.11	-9.86 ± 17.47	5.289	< 0.001
_△_AX(%)	-42.98 ± 25.58	-12.34 ± 24.02	3.535	< 0.001
_△_Fres(%)	-14.19[-25.35, -5.80]	-4.04[-12.56, 1.85]	-4.325	< 0.001

### The diagnostic value of pre-BD IOS pulmonary function parameters in the CVA group

**Fig. 1:**
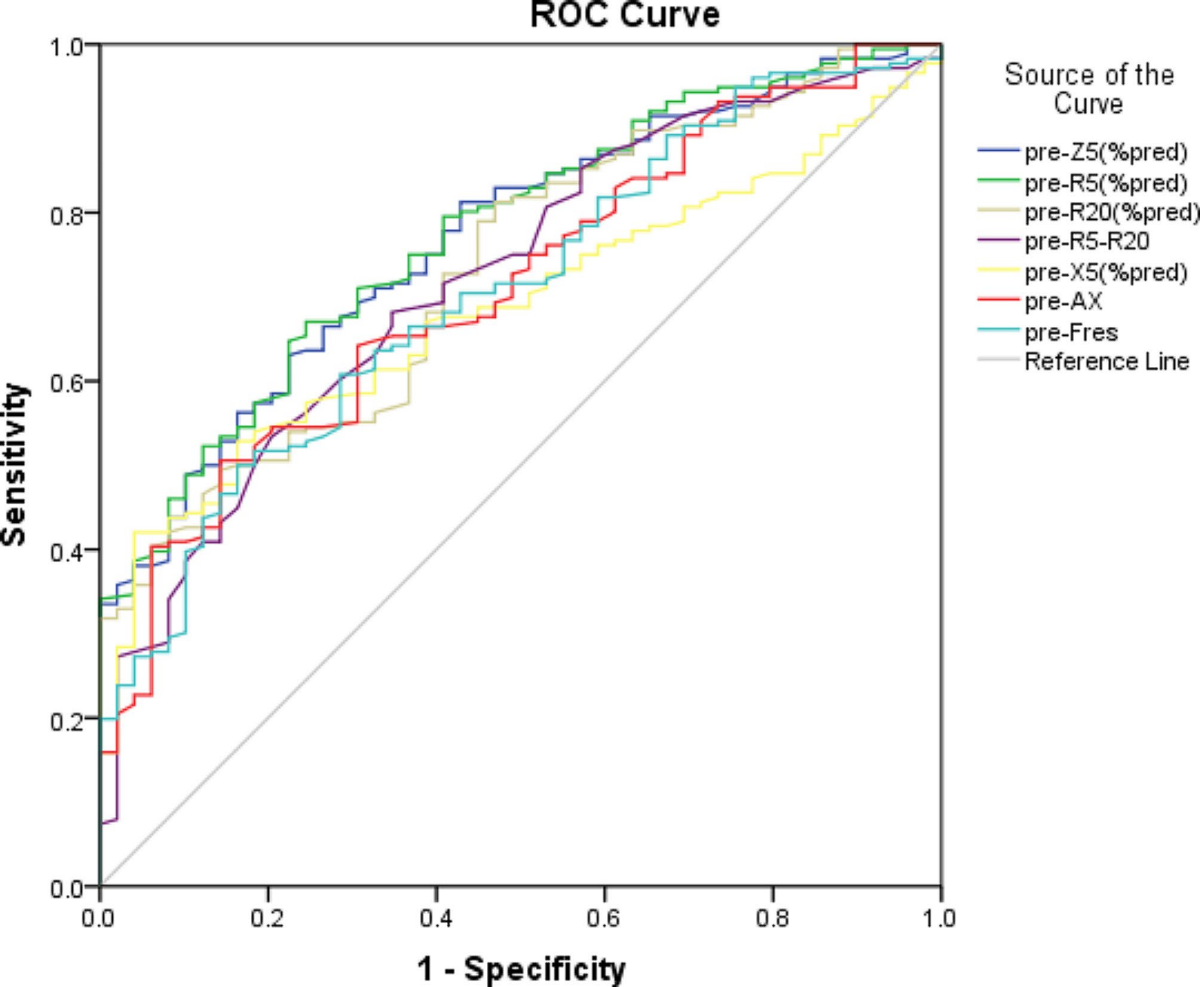
ROC curve for pre-BD IOS pulmonary function parameters in the CVA group

ROC curves were plotted for pre-Z5%pred, pre-R5%pred, pre-R20%pred, pre-R5-R20, pre-X5%pred, pre-AX, and pre-Fres, and the optimal cutoff values of these IOS parameters for discriminating CVA were determined based on the maximum Youden index. Pre-R5%pred achieved the highest AUC value of 0.772, with sensitivity and specificity values of 0.670 and 0.745, respectively. Similarly, pre-Z5%pred (AUC 0.768 sensitivity 0.631, specificity 0.765) also exhibited great performance, followed by pre-R20%pred (AUC 0.741), pre-R5-R20 (AUC 0.722), pre-Fres (AUC 0.712), pre-AX (AUC 0.708), and pre-X5%pred (AUC 0.683) (Table 4; Fig. 1).

**Table 4:** The diagnostic value of pre-BD IOS pulmonary function parameters in the CVA group

	AUC	P	Youden index	cutoff values	sensitivity	specificity
pre-Z5(%pred)	0.768	< 0.001	0.396	99.45	0.631	0.765
pre-R5(%pred)	0.772	< 0.001	0.415	97.05	0.670	0.745
pre-R20(%pred)	0.741	< 0.001	0.362	71.50	0.813	0.549
pre-R5-R20(kPa/(L/s)	0.722	< 0.001	0.240	0.23	0.683	0.557
pre-X5(%pred)	0.683	< 0.001	0.376	123.75	0.415	0.961
pre-AX(kPa/L)	0.708	< 0.001	0.349	2.26	0.506	0.843
pre-Fres(1/s)	0.712	< 0.001	0.343	20.08	0.500	0.843

### The diagnostic value of the improvement rate of each IOS pulmonary function parameter in the CVA group

**Fig. 2:**
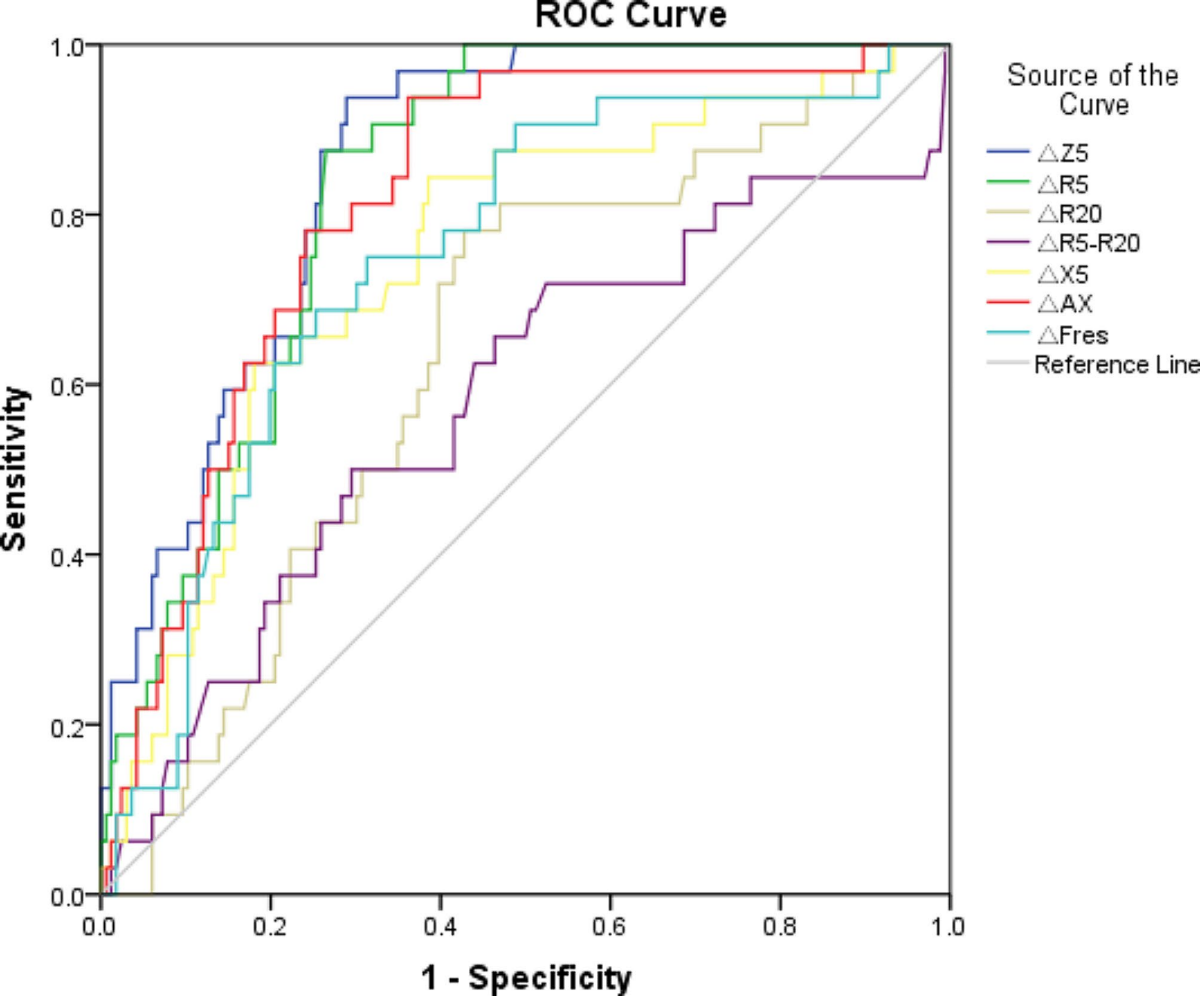
ROC curve for the improvement rate of each IOS pulmonary function parameter in the CVA group

ROC curves were generated for _△_Z5%, _△_R5%, _△_R20%, _△_R5-R20%, _△_X5%, _△_AX%, and _△_Fres%. _△_Z5% achieved the highest AUC value of 0.868. With a threshold value of -16.565%, the sensitivity and specificity of _△_Z5% were 0.959 and 0.706, respectively. This was followed by _△_R5% (AUC 0.843), _△_AX% (AUC 0.824), _△_X5% (AUC 0.748), _△_Fres% (AUC 0.748), and _△_R20% (AUC 0.639) with threshold values of -15.04%, -38.195%, -20.78%, -8.975%, and −14.085%. (Table 5; Fig. 2).

**Table 5:** The diagnostic value of the improvement rate of each IOS pulmonary function parameter in the CVA group

	AUC	P	Youden index	cutoff values	sensitivity	specificity
_△_Z5(%)	0.868	< 0.001	0.665	-16.565	0.959	0.706
_△_R5(%)	0.843	< 0.001	0.593	-15.04	0.860	0.733
_△_R20(%)	0.639	< 0.001	0.454	-14.085	0.865	0.589
_△_R5-R20(%)	0.580	0.151	0.205	-24.17	0.500	0.705
_△_X5(%)	0.748	< 0.001	0.440	-20.78	0.824	0.616
_△_AX(%)	0.824	< 0.001	0.576	-38.195	0.943	0.633
_△_Fres(%)	0.748	< 0.001	0.339	-8.975	0.667	0.672

**Fig. 3:**
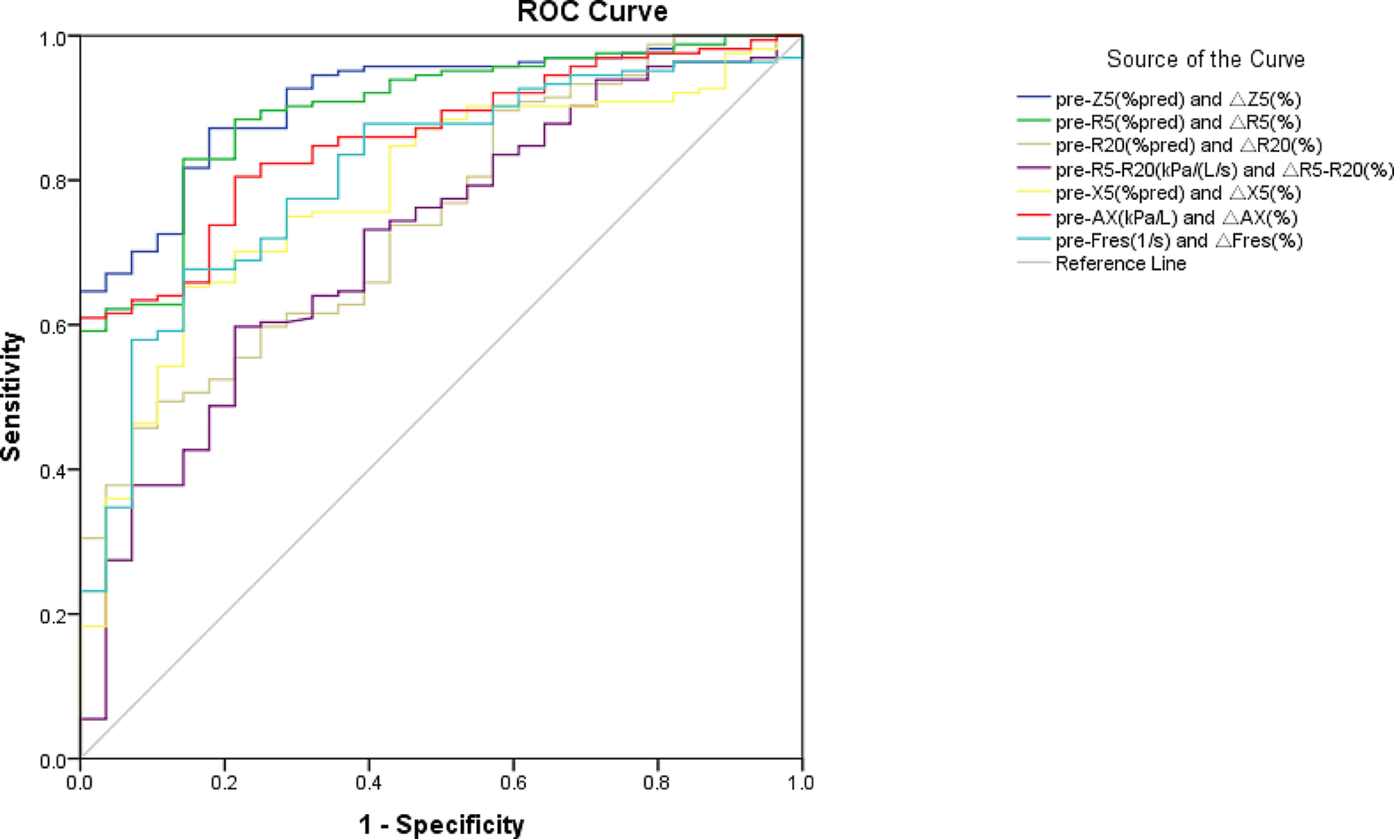
The combined ROC curve for pre-BD and the improvement rate of IOS parameter in the CVA group

The ROC curves for combinations of pre-BD parameters and their corresponding improvement rates during the BD test were also assessed. Notably, the combination of “pre-Z5%pred and _△_Z5%” (AUC 0.920, sensitivity 0.877, specificity 0.833) and “pre-R5%pred and _△_R5%” (AUC 0.898, sensitivity 0.830, specificity 0.867) demonstrated excellent performance. Following in performance were pre-AX combined with _△_AX% (AUC 0.867), pre-Fres combined with _△_Fres% (AUC 0.815), and pre-X5%pred combined with _△_X5% (AUC 0.796) (Table 6; Fig. 3).

**Table 6:** The diagnostic value of the combination of pre-BD and the improvement rate of IOS parameter in the CVA group

Combinations	AUC	P	Youden index	cutoff values	sensitivity	specificity
pre-Z5(%pred) and _△_Z5(%)	0.920	< 0.001	0.710	90.7%;-14.02%	0.877	0.833
pre-R5(%pred) and _△_R5(%)	0.898	< 0.001	0.697	81.1%;-19.90%	0.830	0.867
pre-R20(%pred) and _△_R20(%)	0.758	< 0.001	0.397	88.7%;-9.64%	0.497	0.900
pre-R5-R20(kPa/(L/s) and _△_R5-R20(%)	0.716	< 0.001	0.384	0.21;-28%	0.598	0.786
pre-X5(%pred) and _△_X5(%)	0.796	< 0.001	0.528	121.5%;-16.25%	0.661	0.867
pre-AX(kPa/L) and _△_AX(%)	0.867	< 0.001	0.614	2.19;-41.29%	0.614	0.960
pre-Fres(1/s) and _△_Fres(%)	0.815	< 0.001	0.540	16.89;-25.44%	0.673	0.867

## Discussion

This study investigated the diagnostic utility of IOS parameters for identifying pediatric CVA. _△_Z5 demonstrated the highest performance among the individual IOS parameters, with an AUC of 0.868. Furthermore, the combined parameters pre-Z5%pred+_△_Z5%, pre-R5%pred+_△_R5%, and pre-AX+_△_AX% surpassed the other combinations, exhibiting superior diagnostic performance for CVA with AUCs of 0.920, 0.898, 0.867, respectively.

Currently, the diagnostic criteria for CVA in children are not fully specified [5, 12]. The generally accepted standard for diagnosing classic asthma involves an improvement rate of FEV_1_%pred ≥ 12% following anti-asthmatic therapy or bronchodilator inhalation. However, FEV_1_ in CVA patients is often near normal, making _△_FEV_1_%pred ≥ 12% less suitable for CVA diagnosis [23]. Furthermore, FEV_1_ provides a limited assessment of the airway, predominantly reflecting the airflow limitation in medium and large airways [24]. Recent studies suggest IOS outperforms spirometry in the early detection of mild reversible airway obstruction [18, 25, 26]. IOS is commonly employed to passively measure the mechanical properties of the respiratory system, particularly in pediatric cases where cooperation for spirometry is challenging. Among the parameters of IOS, R5 reflects the whole airway resistance, while R20 and R5-R20 represent the central and peripheral airway resistance, respectively. Small airway parameters also include X5, AX, and Fres [18, 27, 28].

Research on IOS in children with classic asthma has demonstrated its high sensitivity and specificity, particularly in children aged 3 to 4 years. A significant decrease of at least 20% in Zrs and R5, as well as a 30% increase in X5 after bronchodilator inhalation, are reliable indicators for diagnosing asthma [29]. Moreover, IOS in children with classic asthma showed that _△_X5 and _△_AX could detect mild reversible airway obstruction earlier than spirometry [18]. However, the diagnostic value of IOS parameters and their changes during BD tests in children with CVA has not been clarified. Our study observed that the CVA group exhibited more significant improvement in all IOS parameters than the control group. Among the individual IOS parameters, _△_Z5% (AUC 0.868) had the highest diagnostic performance, followed by _△_R5% (AUC 0.843) and _△_AX% (AUC 0.824). When considering combined parameters, pre-Z5%pred+_△_Z5%, pre-R5%pred+_△_R5%, and pre-AX+_△_AX% achieved the highest AUCs of 0.920, 0.898, and 0.867, respectively. These parameters accurately identified a significant percentage of children with CVA, indicating excellent performance in identifying CVA in children. These findings emphasize the importance of considering changes in IOS parameters during BD tests to diagnose CVA. Furthermore, we provide precise threshold values for these parameters to aid in differentiating between children with and without CVA.

Previous adult studies have demonstrated that mucus clogging and inflammatory lesions are present in both small and large airways in lung biopsy pathology of patients with chronic asthma and in autopsy pathology of patients with fatal asthma [30–32]. Classic asthma is characterized not only by large airway dysfunction but also by small airway dysfunction [33]. CVA shares pathophysiologic characteristics with classical asthma, such as eosinophilic airway inflammation but less airway remodeling [34, 35]. CVA manifests as small airway spasms without significant large airway dysfunction, and spirometry shows small airway changes [15]. However, changes in the large and small airways in children with CVA have yet to be thoroughly elucidated [36]. In this study, among all pre-IOS parameters in children with CVA, X5 was found to be lower than that in the control group, while the remaining parameters were significantly higher, indicating elevated peripheral airway elastic resistance and total respiratory resistance in children with CVA. After inhaling salbutamol sulfate, the differences in most small airway parameters (R5-R20, X5%pred, AX, and Fres ) between the two groups were not significant, indicating that the peripheral airway obstruction was reversible. However, Z5%pred, R5%pred, and R20%pred during the BD test did not return to normal levels, raising the possibility of early central airway remodeling in children with CVA. Further analysis revealed that 29.4% of the changes in the central airway in our study were irreversible.

A study investigating the relationship between IOS parameters and the effectiveness of ICSs in adults with CVA revealed that patients with peripheral airway obstruction responded better to fine-grain ICSs than to coarse-grain ICSs. Conversely, individuals with central airway obstruction exhibited better responses to coarse-grain ICSs [25]. It would be intriguing to investigate whether these ICSs demonstrate varying effectiveness in children with CVA with central or peripheral airway dysfunction as detected by IOS, potentially offering valuable guidance for CVA treatment in the future.

Our study has several limitations that should be noted. Firstly, it was conducted at a single center with a relatively small sample size of CVA patients and an even smaller control group, potentially limiting the generalizability of our results. Multicenter and large sample studies should be considered for future research. Secondly, our study encompassed children aged 4 to 12 years, lacking insights into how CVA affects IOS pulmonary function in children under four years old. Moreover, we did not include an analysis of FeNO measurements, which were recognized as important contributors when combined with IOS parameters for distinguishing CVA in preschool children [20]. Future studies should investigate whether FeNO levels also enhance the discrimination ability in CVA children of older age groups. Finally, our control group was comprised solely of healthy children, which might not entirely represent the entire population. Future studies would include non-CVA children with chronic cough as a control group to better characterize the differences between children with CVA and children with other respiratory diseases.

## Conclusion

The IOS parameters were found to be normal or nearly normal in children with CVA, yet significantly greater than those in healthy children. The changes in IOS parameters during the BD test provided valuable diagnostic information for CVA. Despite the limitations above, our study presents new possibilities and insights for enhancing the diagnosis of CVA in children.

## Data Availability

The original data used in this study are available from the corresponding author upon reasonable request.

## Abbreviations

AUC: Area under the ROC curve
AX: Integrated area of low-frequency X
BD: Bronchodilation
BHR: Bronchial hyperresponsiveness
BPT: Bronchial provocation test
Fres: Resonant frequency
FeNO: Fractional exhaled nitric oxide
GINA: The Global Initiative for Asthma
ICS: Inhaled corticosteroid
IOS: Impulse oscillometry
R5: Resistance at 5 Hz
R20: Resistance at 20 Hz
ROC: Receiver operating characteristic
X5: Reactance at 5 Hz
Z5: Respiratory impedance at 5 Hz
